# Water Level Regulation Regime Shifts Drive Divergent Foraging Habitat Use by Wintering Hooded Cranes (*Grus monacha*) in Shallow Gate‐controlled Lakes of the Yangtze Floodplain

**DOI:** 10.1002/ece3.73800

**Published:** 2026-06-09

**Authors:** Yong Fang, Yundong Zhong, Lizhi Zhou, Ming Liang

**Affiliations:** ^1^ School of Resources and Environmental Engineering Anhui University Hefei China; ^2^ Anhui Province Key Laboratory of Wetland Ecosystem Protection and Restoration Anhui University Hefei China; ^3^ Anhui Shengjin Lake Wetland Ecology National Long‐term Scientific Research Base Dongzhi China

**Keywords:** artificial habitats, foraging flock, gate‐controlled lakes, habitat use, hooded crane, water level regulation

## Abstract

Habitat use is fundamental for animal survival at key life history stages. Understanding how changes in foraging habitat conditions modulate habitat‐use patterns is critical in wildlife conservation. For wintering waterbirds, habitat use is tightly coupled with food abundance and availability, both of which are highly sensitive to water‐level fluctuations. The Hooded Crane ( *Grus monacha* ) is a flagship wintering wader species found in shallow lakes of the middle‐lower Yangtze River floodplain. However, hydraulic gate‐driven artificial water level regulation has modified food available in foraging habitats, prompting urgent research on species‐specific adaptive habitat utilization strategies. From November 2024 to March 2025, we surveyed the foraging population size and distribution dynamics of Hooded Cranes at Shengjin and Caizi lakes, which are two shallow lakes with contrasting water management regimes. We quantified the relationship between water levels and available habitat area for Hooded Crane, analyzed habitat‐use patterns and preferences, and used Generalized Linear Mixed Models to assess environmental drivers of foraging abundance. The results showed that water level fluctuations influenced natural habitat availability. Habitat use patterns diverged markedly between lakes: At Shengjin Lake, cranes strongly preferred mudflats (75.47% of foraging observations), with abundance driven by food biomass and sediment penetrability (*p* < 0.001), supporting the predictions of Optimal Foraging Theory. Conversely, cranes at Caizi Lake relied predominantly on paddy fields (75.97%). Their abundance was co‐determined by food biomass and road proximity. Such patterns align with Risk‐Sensitive Foraging Theory, revealing that cranes accept greater human interference to fulfill energy needs as natural habitats shrink. While temporarily buffering food shortages, this habitat shift increases potential survival costs. This study provides a mechanistic basis for adaptive management trade‐offs in gate‐controlled wetlands and offers critical guidance for conserving endangered wintering waterbirds in the gate‐controlled lakes in Yangtze floodplain.

## Graphical abstract

This study investigates how contrasting hydrological regulation regimes at two gate‐controlled lakes influence the foraging strategies of wintering Hooded Cranes. We demonstrate that while cranes at near‐natural sites follow Optimal Foraging Theory by utilizing high‐accessibility mudflats, those at artificially high‐water sites exhibit an adaptive shift toward a Risk‐Sensitive Foraging strategy, exploiting energy‐rich artificial habitats despite increased anthropogenic disturbance. Our findings highlight the behavioral plasticity of waterbirds in response to human‐induced habitat loss and emphasize the critical role of artificial landscapes as functional substitutes in degraded wetland ecosystems.

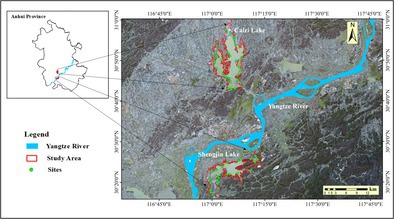

## 1 Introduction

Habitat use is a fundamental survival strategy for animals. Therefore, understanding the spatial distribution of wildlife is essential for effective conservation and management (Kong et al. 2018). According to the Optimal Foraging Theory (OFT), animals select the most efficient and profitable foraging habitats to maximize energy intake while minimizing costs (Fan et al. 2020; Davis et al. 2022). However, OFT models often assume predictable environments, whereas in the wild, food resources are frequently variable and uncertain (Houston and Rosenström 2024). Risk‐Sensitive Foraging Theory (RSFT) complements this by positing that foraging choices depend not only on the expected reward but also on the variability and predictability of food options (Banerjee and Thaker 2025).

However, the global intensification of anthropogenic disturbance has altered animal habitat‐use patterns substantially (Zhou et al. 2021). Adaptive adjustments in habitat use can help mitigate the negative effects of environmental change (Wachu et al. 2025). For wintering waterbirds, habitat use is particularly dependent on wetland hydrological regimes (Deng et al. 2023). Natural water level fluctuations create suitable habitats with abundant and predictable resources (Wang et al. 2024). In contrast, artificial regulation through hydraulic gates severely degrades natural habitats, altering the availability and predictability of food (Guo et al. 2022; Shen et al. 2025). Consequently, waterbirds must modify their foraging habitat‐use patterns to cope with such altered conditions and secure sufficient food resources (Cui et al. 2023; Deng et al. 2023).

Habitat use is a complex decision‐making process involving trade‐offs between benefits and risks, under the influence of multiple factors (Fan et al. 2020; Zhao et al. 2013). Food abundance and availability are considered the primary drivers (Bi and Zhou 2025; Ucero et al. 2025). In recent years, intensive human activities have degraded the quality of traditional foraging habitats for wintering waterbirds progressively (Wang et al. 2026). When food resources in such habitats become insufficient, animals may be forced to seek alternative habitats to alleviate increased survival risk (Xu et al. 2020). However, the quality of alternative habitats is generally low, particularly because paddy fields are subject to heightened human activity, which can disrupt foraging success considerably (Harrison et al. 2018). Although animals possess considerable adaptive capacity under habitat change (Zhu et al. 2021), alternative habitats are not necessarily optimal and may impose increased survival costs, such as reduced food intake and lower foraging efficiency (Yang et al. 2015; Wu et al. 2020). Therefore, investigating habitat use patterns under anthropogenic disturbance is crucial for effective conservation management.

The middle and lower Yangtze River floodplain has the highest density of lakes in China, providing critical wintering and stopover sites for migratory waterbirds on the East Asian‐Australasian Flyway (Wei and Zhou 2023). Shallow river‐connected lakes in the region are regulated extensively by gates and dams (Wang et al. 2017). These hydraulic structures are typically closed during the rainy season and opened during the dry season to release water, thereby maintaining near‐natural water‐level fluctuations that provide suitable habitats for wintering waterbirds (Cui et al. 2024). However, driven by the demands for flood control, drought mitigation, and water storage, the hydrological regimes of certain lakes have undergone significant alterations. In particular, as a key lake in the region, Caizi Lake is now maintained at significantly higher water levels during the wintering period. This persistent high‐water regulation disrupts the lake's inherent drawdown rhythm, causing the prolonged inundation of mudflats and extensive natural habitat loss (Yao et al. 2022; Cui et al. 2024), while simultaneously suppressing the growth of submerged macrophytes such as *Vallisneria* spp. (Zhi et al. 2018). These changes severely weaken the capacity of such lakes to support herbivorous wintering waterbirds (Guan et al. 2014; Wu et al. 2021), thereby altering the foraging habitat‐use patterns of species that depend on such ecosystems (Cheng et al. 2022).

Hooded Crane ( *Grus monacha* ) is a vulnerable (VU) species on The International Union for Conservation of Nature (IUCN) Red List and a key herbivorous waterbird wintering in the middle and lower Yangtze River wetlands. Shengjin and Caizi Lakes serve as core wintering sites, supporting nearly two‐thirds of the population in China (Zheng et al. 2015). The distribution of Hooded Cranes in these lakes is closely coupled with the exposure of submerged macrophyte tubers, such as *Vallisneria natans* and *Potamogeton wrightii*, as well as the accessibility of mollusks (Huang et al. 2018). However, due to progressive wetland degradation and the alteration of natural hydrological rhythms caused by gates and dams, the supply of food resources within these lakes has declined significantly. Since the 1950s, local residents have used polders to establish paddy fields for expanding arable land; the residual grains remaining after harvest provide an accessible food resource for Hooded Cranes (Cheng et al. 2022). Consequently, Hooded Cranes have developed greater foraging flexibility in their habitat use within these lakes. Recent studies indicate that these cranes increasingly exploit paddy fields to compensate for the food shortages in natural wetlands (Cui et al. 2023).

Despite growing recognition of hydrological alterations' impacts on waders, the mechanistic link between contrasting water‐level regulation regimes and the adaptive foraging trade‐offs of Hooded Cranes remains unclear. To address this gap, we surveyed the spatiotemporal foraging dynamics of Hooded Cranes at Shengjin Lake (near‐natural water level fluctuation) and Caizi Lake (artificially maintained high water levels), testing two key hypotheses. First, the hydrological regulation regimes of gate‐controlled lakes directly determine the availability of natural foraging habitats. We hypothesize that prolonged high‐water levels lead to the physical loss of natural habitats, triggering an adaptive spatial shift in Hooded Cranes and establishing artificial habitats (paddy fields and aquaculture ponds) as essential functional substitutes for population maintenance. Second, while food resources remain the primary driver of population abundance and distribution, foraging trade‐offs are mediated by local environmental constraints. Specifically, we hypothesize that at Shengjin Lake, foraging follows OFT, prioritizing high‐accessibility, low‐disturbance natural habitats. Conversely, at Caizi Lake, cranes follow RSFT, tolerating heightened anthropogenic disturbance in exchange for high energy rewards in artificial habitats.

## 2 Materials and Methods

### 2.1 Study Area

Shengjin (116°55′–117°15′ E, 30°15′–30°30′ N) and Caizi (116°58′–117°11′ E, 30°43′–30°59′ N) lakes are situated in southern Anhui Province, China. Both regions experience a northern subtropical humid monsoon climate characterized by four distinct seasons, abundant sunshine, and rainfall. Shengjin Lake consists of three interconnected sub‐lakes (the Upper, Middle, and Lower lakes), with an annual average temperature of 16.1°C (summer average: 28.8°C; winter average: 3.9°C) and average annual precipitation of 1600 mm (Xu et al. 2022). Caizi Lake comprises three connected sections (Baitu, Caizi, and Xizi lakes), with an annual mean temperature of 16.5°C (summer: 29.0°C; winter: 3.5°C) and annual precipitation ranging from 1200 to 1389 mm (Zheng et al. 2022).

**FIGURE 1:**
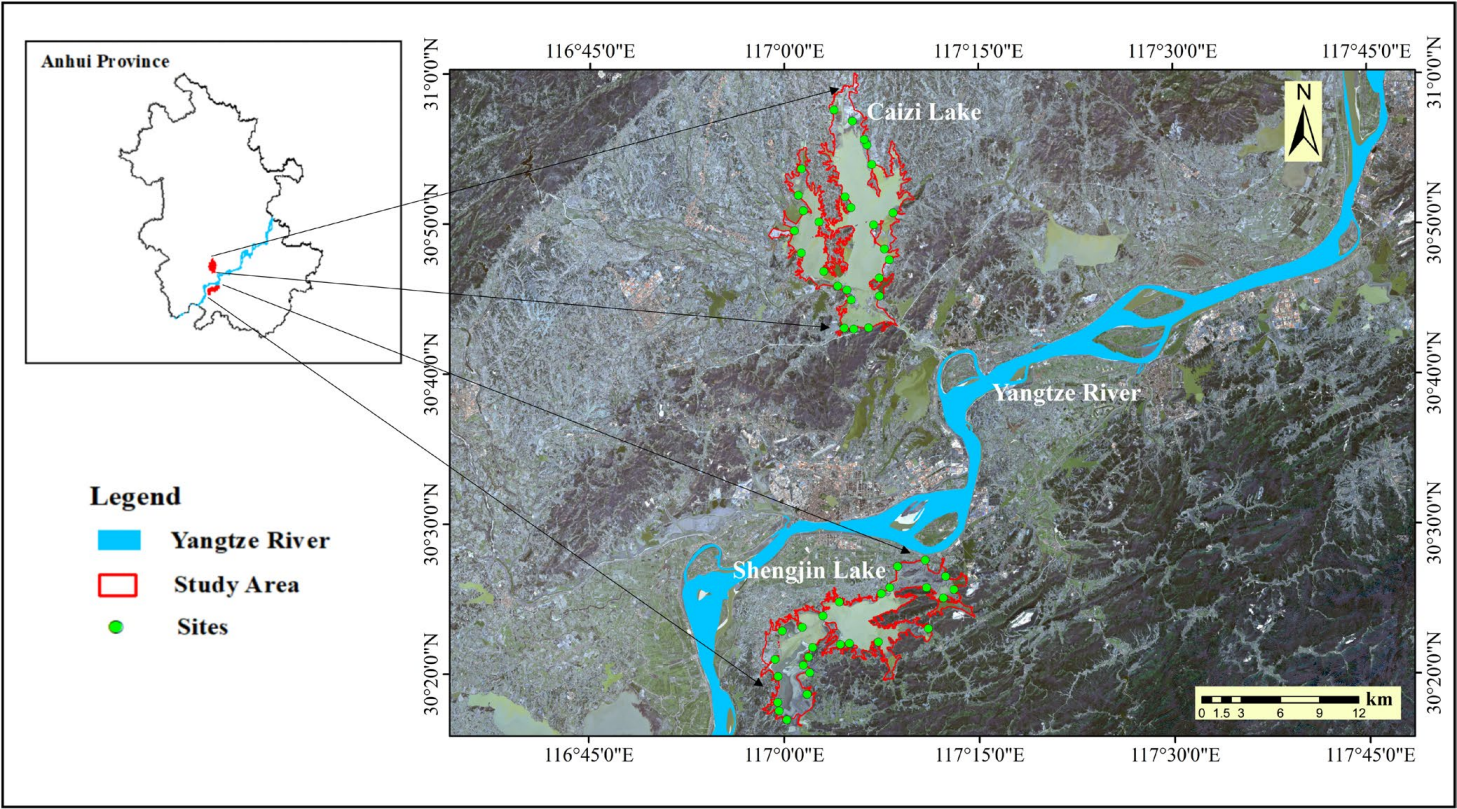
Study area and survey sites at Shengjin and Caizi lakes, located at the border of Anqing, Tongling, and Chizhou cities in Anhui Province, China. The remote sensing interpretation of lake habitats is based on satellite imagery from January 15, 2025, during the low‐water period.

The two lakes have high similarities in wetland hydrology and ecological processes, with their water levels regulated by the Huangpen and Zongyang gates, respectively (Figure 1). Both exhibit periodic seasonal water level fluctuations. Historically, the average water level of Shengjin Lake was 12.69 m (All water levels adopt the National Vertical Datum 1985) during the rainy season (May–September) and 9.73 m during the dry season (October–April) (Zhang et al. 2021). For Caizi Lake, the multiyear average water level during the dry season is 8.67 m, with a mean difference of 3.34 m between the rainy and dry seasons (Wang et al. 2018; An et al. 2021). Near‐natural seasonal water level fluctuations facilitate the exposure of mudflats and promote plant growth, thereby providing ample habitat and food resources for wintering waterbirds (Wang et al. 2024). However, in recent years, significant discrepancies in water‐level fluctuations have emerged between the two lakes. Specifically, Caizi Lake has maintained significantly higher water levels, which has reduced the area of suitable habitats available for wintering migrants, inhibited the growth of wetland vegetation, and consequently negatively impacted wintering waterbird growth and development (Cheng et al. 2022; Cui et al. 2024).

### 2.2 Water Level Data and Habitat Classification

**FIGURE 2:**
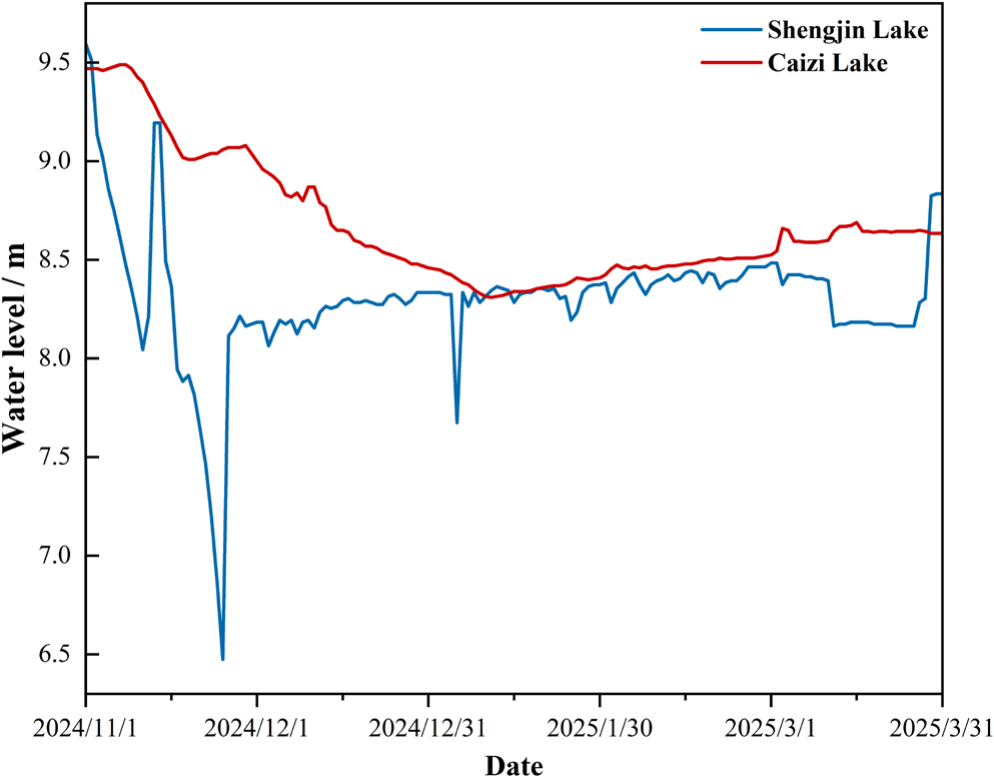
Daily water level trends in Shengjin and Caizi lakes from November 2024 to March 2025.

Daily water level data recorded at 08:00 am from November 2024 to March 2025 were retrieved from the Anhui Provincial Hydrological Information Network (http://yc.wswj.net/ahsxx/LOL/?refer=upl&to=public_public). All water levels were referenced to the 1985 National Elevation Datum (Figure 2). Furthermore, to characterize the divergent water level regulation regimes between the two lakes, we extended our analysis by collecting daily water level data during the wintering period (December to February) from 2014 to 2025. Monthly mean water levels were then calculated for this 11‐year period to identify long‐term hydrological trends and potential regime shifts. These historical data provide a comparative baseline to determine how anthropogenic gate regulation or natural hydrological variations have altered the foraging habitat availability for wintering cranes over the past decade (Figure S1).

Sentinel‐2 images of the study area were downloaded from the ESA Copernicus Data Center (https://browser.dataspace.copernicus.eu/). A total of 20 images were selected for subsequent land cover classification, all meeting two key criteria: cloud cover < 10%, and water levels on the image acquisition dates closely aligned with the monthly mean levels of the respective lakes. The vector boundaries of the study areas were defined based on the maximum water levels recorded at the Shengjin and Caizi lakes over the preceding five years. Additionally, the boundaries of artificial foraging habitats (including paddy fields and aquaculture ponds) were delineated through field surveys using Google Earth Pro (Google, Mountain View, CA, USA). The lake areas were classified into three categories: water, mudflats, and meadows, using the Maximum Likelihood Classification algorithm. A class separation distance > 1.9 was maintained to ensure classification reliability. A confusion matrix was constructed to assess classification accuracy, followed by post‐classification processing to compute habitat statistics. The accuracy values derived from the confusion matrix were all > 90%, with a kappa coefficient > 0.9, indicating high classification accuracy (Elgin et al. 2020). All remote sensing classifications and accuracy assessments were performed using ENVI 5.6 (NV5 Geospatial., Broomfield, CO, USA).

### 2.3 Population and Habitat Surveys

To systematically survey the foraging populations of Hooded Cranes, a 1 × 1‐km grid covering the lake area, surrounding paddy fields, and aquaculture ponds was established. Based on topography, geomorphology, and historical distribution, 26 fixed observation points were established to ensure comprehensive coverage of their foraging habitats (Figure 1). Field surveys were conducted during the wintering period from November 2024 to March 2025. Three comprehensive population surveys were performed monthly for each lake, with each survey conducted from 07:00 to 17:00 (Bi and Zhou 2025). Survey dates were fixed on the 5th–6th, 15th–16th, and 25th–26th of each month; to ensure data synchrony, the comprehensive survey for each lake was completed within a single day. All surveys were carried out under favorable weather conditions; if unfavorable weather such as strong winds, heavy fog, or precipitation occurred on the scheduled dates, the surveys were postponed sequentially. The surveys were conducted simultaneously by two permanent teams of four observers each, which minimized inter‐observer bias. The records were validated when at least two observers confirmed the presence of a crane simultaneously. A foraging habitat use event was recorded when the cranes were observed foraging continuously in a specific habitat for > 15 min (Wu et al. 2020). For each valid event, GPS coordinates, habitat type, and foraging population size were recorded (Figure S2).

At confirmed foraging sites, food resource sampling was conducted within two days following the population surveys (Marshall et al. 2013; Fonseca and Navedo 2020). To avoid flushing the foraging flocks, which would induce unnatural spatial displacement to non‐foraging areas and introduce abnormal bias into the observed distribution patterns, sampling was deliberately avoided during observation periods. Within the identified foraging range, a 1 × 1 km zone was demarcated, where 15 sub‐quadrats (0.5 × 0.5 m) were randomly established to quantify food biomass, burial depth, and sediment penetrability (Zhong et al. 2022). Consequently, these food resource data served as a standardized indicator of short‐term food availability at the observed foraging locations. Using a pointed shovel, pits were excavated to a depth of approximately 15 cm, the approximate reach of a Hooded Crane's beak, to collect vegetative rhizomes and tubers. Primarily, food items included tubers and roots of aquatic macrophytes such as *Vallisneria* spp., *Ranunculus japonicus*, *Potentilla supina* , *Tulipa edulis*, and *Polygonum criopolitanum*, as well as remaining rice grains ( *Oryza sativa* ) in paddy fields and benthic invertebrates in aquaculture ponds (Yang et al. 2015; Hou et al. 2021).

Sediment penetrability (N/cm^2^) was measured adjacent to each sub‐quadrat using a stratometer (TYD‐2; Alisun, Hangzhou, China). Food burial depth was measured using a ruler (Deli, Beijing, China). Samples were transported to the Anhui Shengjin Lake Wetland Ecology National Long‐term Scientific Research Base Laboratory (Dongzhi County, Anhui Province), where they were washed to remove debris and soil and then dried in an oven (YHG‐9050A, Dreip, Suzhou, China) to a constant weight. Dry mass was measured using a laboratory analytical balance with a precision of 0.0001 g (Cui et al. 2023). Food biomass was calculated as the dry mass per quadrat area (g/m^2^) (Bi and Zhou 2025). In total, 145 quadrats were sampled from Shengjin Lake and 72 from Caizi Lake.

### 2.4 Data Analysis and Statistics

Spearman's rank correlation was used to assess the relationship between monthly average water levels and the area of each habitat type in both lakes. The population quantity and habitat utilization frequency of Hooded Cranes were converted into proportional data for subsequent statistical analysis. As the data followed a non‐normal distribution, we used the Kruskal‐Wallis test to evaluate differences in the relative abundance of Hooded Cranes among habitats. For cases with overall significance (*p* < 0.05), Dunn's post hoc test with Holm's correction was applied for multiple pairwise comparisons. Ivlev's electivity index (*S*_*i*_) was used to evaluate monthly habitat preferences. The index is calculated as follows:

$$S_{i}=\frac{\left(a_{i}-b_{i}\right)}{\left(a_{i}+b_{i}\right)}$$

where *a*_*i*_ represents the proportion of the crane population using habitat *i* and *b*_*i*_ represents the proportion of the area of habitat *i* relative to the total available habitat, derived from monthly remote sensing interpretation (Kong et al. 2018). Values range from −1 to +1, where negative values indicate avoidance, zero indicates random use, and positive values indicate preference for that habitat type (Lee et al. 2025). To characterize habitat preferences over specific periods, Ivlev's electivity indices obtained from multiple surveys were averaged and expressed as mean ± SE.

Generalized Linear Mixed Models (GLMMs) were developed separately for each lake to quantify the environmental drivers of foraging population abundance (Xu et al. 2024). Population abundance was modeled as a response variable. Fixed effects included food biomass, burial depth, sediment penetrability, distance to roads, and distance to settlements, was set as fixed effects. Foraging habitat ID and survey sessions were included as random effects. A negative binomial distribution was specified. The predictor variables were standardized for further analysis. We assessed multicollinearity among independent variables using the variance inflation factor (VIF) test, with the diagnostic threshold set at 5 (Liu et al. 2024). Model selection was based on the second‐order Akaike Information Criterion (AICc) for small sample sizes and Akaike weights. Models with ∆AICc < 2 were retained as candidate models. Parameter *p*‐values and 95% confidence intervals were estimated using the Wald test. The relative importance of each variable was estimated by summing the Akaike weights across all models containing that variable. All statistical analyses were performed using R v4.5.1 (R Foundation for Statistical Computing, Vienna, Austria).

## 3 Results

### 3.1 Relationship Between Water Level and Foraging Habitat Area

Spearman's correlation analysis revealed distinct patterns between the two lakes. At Shengjin Lake, water levels were significantly positively correlated with water area (*R* = 1.00, *p* < 0.001) and significantly negatively correlated with mudflat area (*R* = −0.9, *p* = 0.037), whereas a moderate but statistically non‐significant negative correlation was observed with meadow area (*R* = −0.70, *p* = 0.188). For Caizi Lake, water levels exhibited a significant positive correlation with water area (*R* = 1.00, *p* < 0.001), a significant negative correlation with mudflat area (*R* = −1.00, *p* < 0.001), and no significant correlation with meadow area (*R* = −0.20, *p* = 0.747).

**FIGURE 3:**
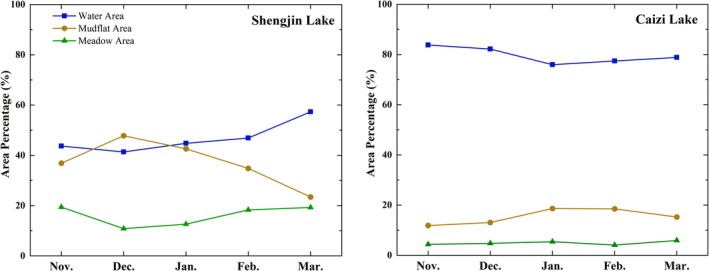
Changes in the relative wintering habitat area at Shengjin and Caizi lakes from November 2024 to March 2025.

Owing to continuous water level fluctuations at Shengjin Lake, the relative area of available natural foraging habitats (mudflats and meadows) exceeded 50% of the total lake area in all months excluding March. In contrast, Caizi Lake maintained high and stable water levels, with the relative water area accounting for over 75% of the total lake area. Consequently, the availability of natural foraging habitats at Caizi Lake was significantly lower than that at Shengjin Lake (Figure 3).

### 3.2 Spatiotemporal Distribution Dynamics of Foraging Populations

**FIGURE 4:**
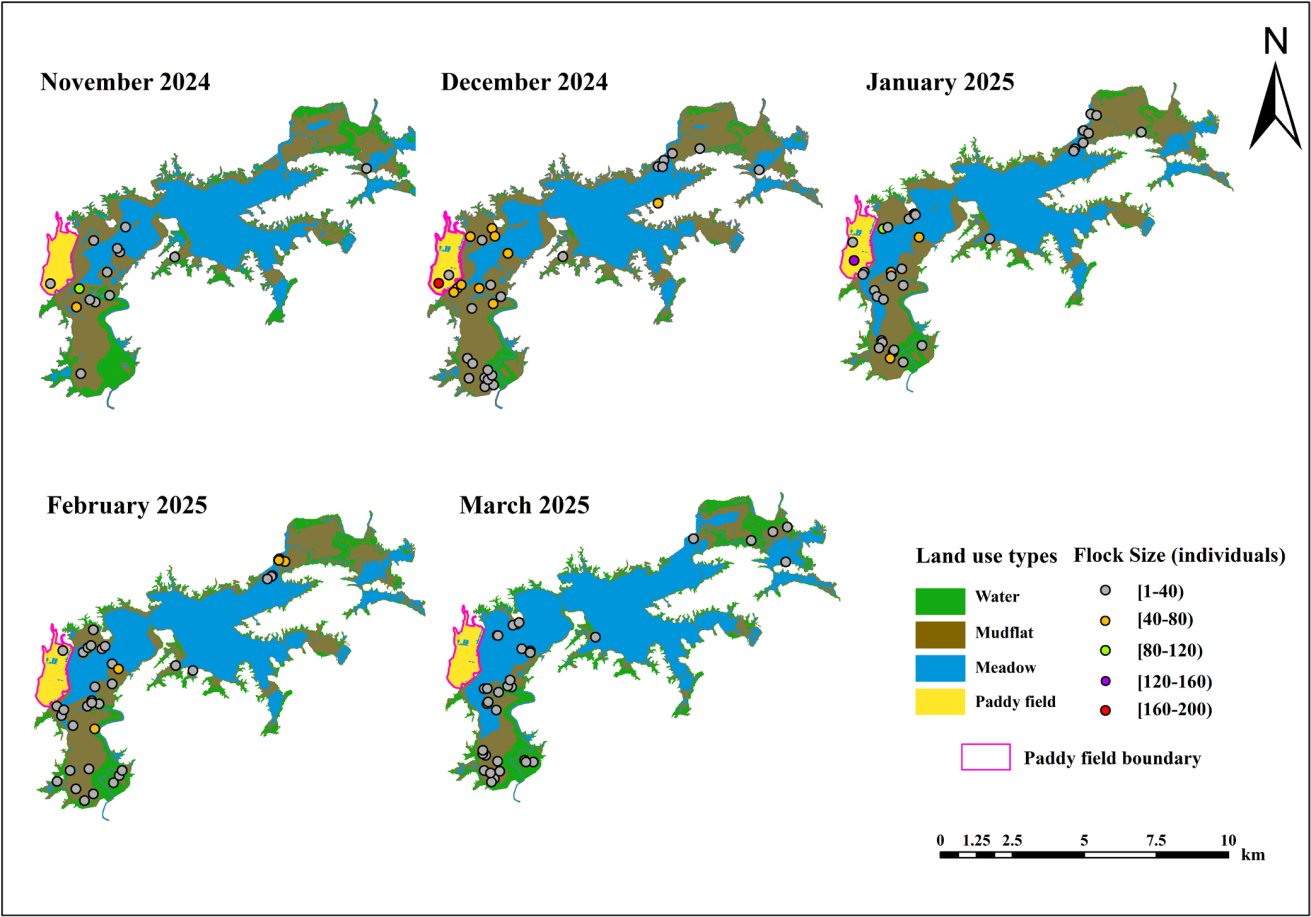
The foraging flocks of wintering Hooded Crane at Shengjin Lake from November 2024 to March 2025.

**FIGURE 5:**
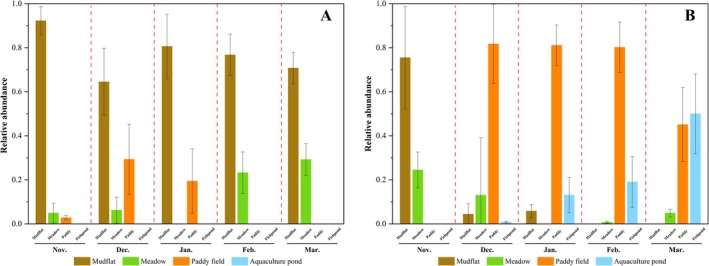
Relative abundance (mean ± SE) of the Hooded Crane populations across different foraging habitats at Shengjin (A) and Caizi (B) lakes.

**FIGURE 6:**
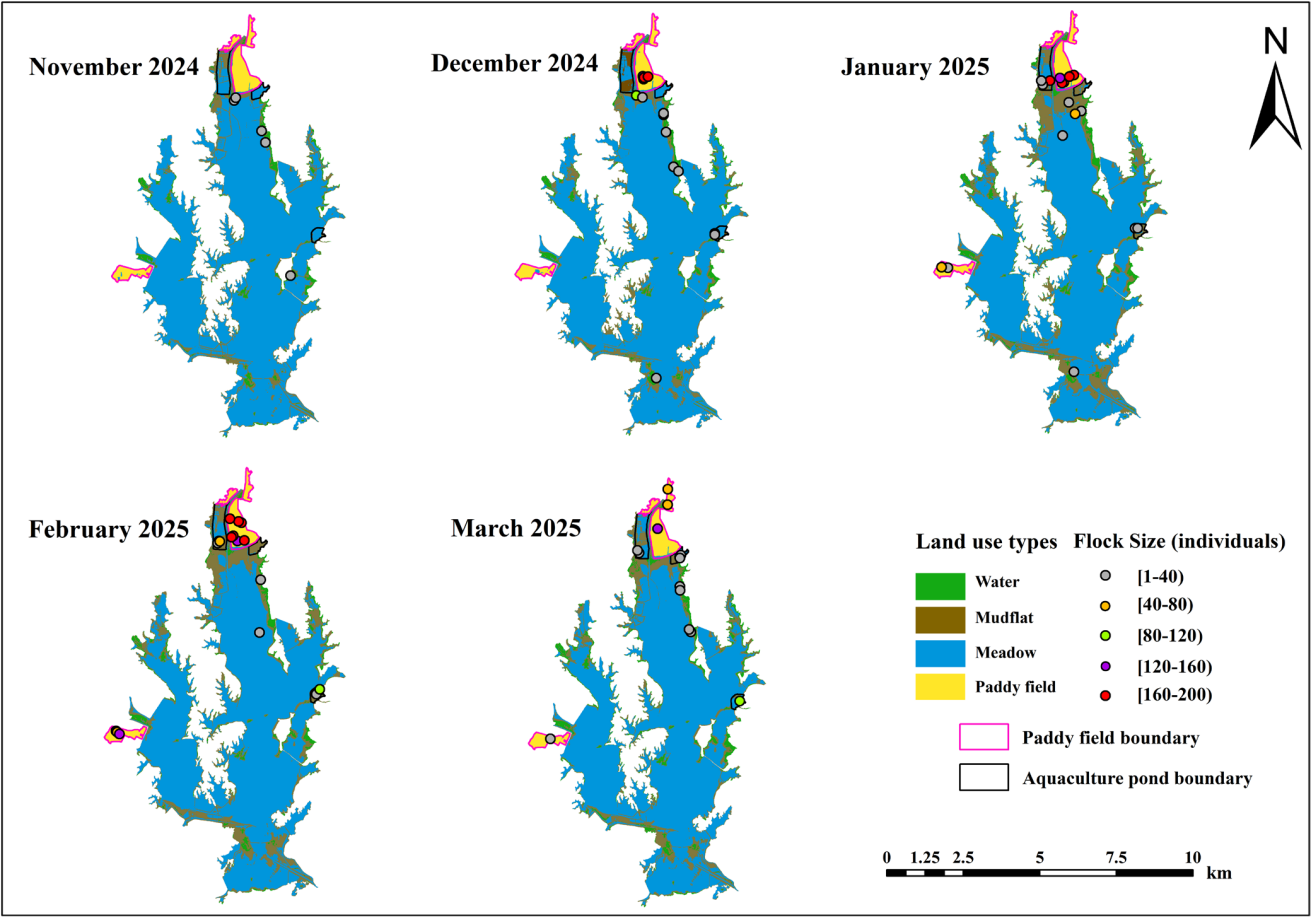
The foraging flocks of wintering Hooded Crane at Caizi Lake from November 2024 to March 2025.

At Shengjin Lake, the foraging population of Hooded Cranes was concentrated in the mudflats of the southern lake area (Figure 4), which supported the highest relative abundance (75.47%, *n* = 15), followed by paddy fields at 12.74% and meadows at 11.79% (Figure 5). The maximum mean foraging population size was recorded in mudflat habitats during January (207.67 ± 97.44 individuals). In contrast, Hooded Cranes at Caizi Lake were concentrated in paddy fields and aquaculture ponds outside the northeastern lake area (Figure 6). Among the habitats, paddy fields supported the highest relative abundance at 75.97%, followed by aquaculture ponds at 17.74%, while mudflats at 3.53% and meadows at 2.75% were rarely used (Figure 5). The largest mean foraging population was observed in paddy fields in February (421.00 ± 96.03 individuals).

**FIGURE 7:**
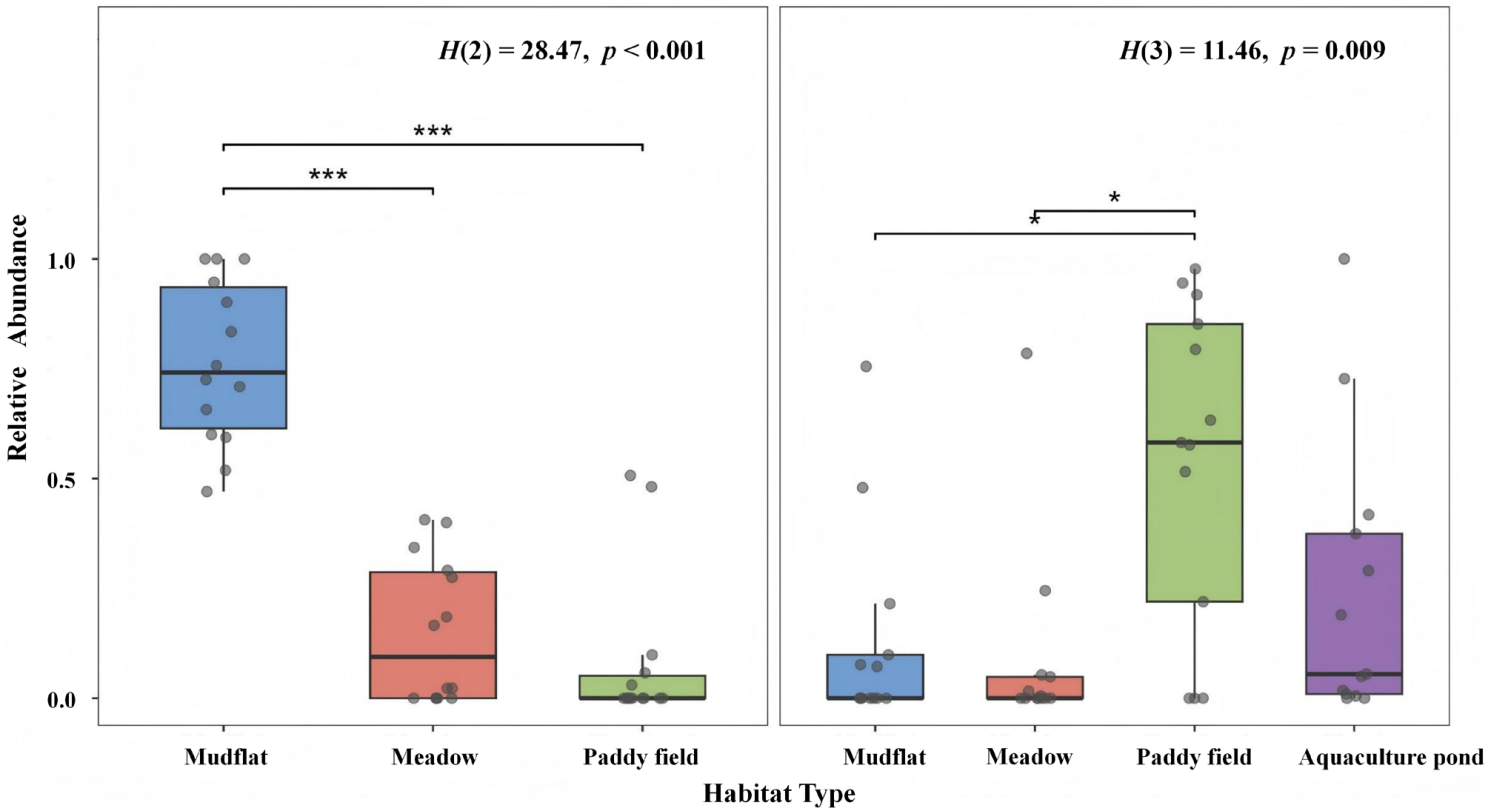
Differential relative abundance of Hooded Cranes across foraging habitats at Shengjin and Caizi lakes. *p* values indicate statistical significance (*0.01 < *p* ≤ 0.05; **0.001 < *p* ≤ 0.01; ***p ≤ 0.001).

There were significant differences in relative abundance among habitats at Shengjin Lake (*χ* ^2^ = 28.47, df = 2, *p* < 0.001; Figure 7). Specifically, the abundance in mudflats was significantly higher than that in both paddy fields (*p* < 0.001) and meadows (*p* < 0.001), whereas no significant difference was detected between paddy fields and meadows (*p* = 0.423). Similarly, significant differences in relative abundance among habitats were found at Caizi Lake (*χ* ^2^ = 11.46, df = 3, *p* = 0.009): the abundances in paddy fields were significantly higher than in meadows (*p* = 0.014) and mudflats (*p* = 0.043), while no significant differences were observed among the other habitat combinations (*p* > 0.05).

### 3.3 Foraging Habitat Use and Influencing Factors

Foraging habitat usage patterns differed significantly between the two lakes (Table 1). At Shengjin Lake, Hooded Cranes showed high stability in the use of lake habitats. The combined utilization frequency of mudflats and meadows consistently exceeded 90% throughout the wintering period and reached 100% in February and March, whereas the use frequency of paddy fields peaked at only 7.69%. At Caizi Lake, foraging habitat use exhibited a distinct seasonal shift where the utilization of mudflats and meadows declined continuously, dropping to a minimum frequency of 11.11% in February, with no use of mudflats in February and March. Meanwhile, the use frequency of paddy fields and aquaculture ponds increased, peaking at 55.56% in February and 73.33% in March, respectively.

**TABLE 1:** Foraging habitat use of overwintering hooded cranes at Shengjin and Caizi lakes.

Lakes	Months	Mudflat (%)	Meadow (%)	Paddy field (%)	Aquaculture pond (%)
Shengjin Lake	Nov.	76.92	15.38	7.69	0.00
	Dec.	78.13	15.63	6.25	0.00
	Jan.	93.94	0.00	6.06	0.00
	Feb.	82.35	17.65	0.00	0.00
	Mar.	71.88	28.13	0.00	0.00
Caizi Lake	Nov.	80.00	20.00	0.00	0.00
	Dec.	50.00	7.14	28.57	14.29
	Jan.	25.00	0.00	50.00	25.00
	Feb.	0.00	11.11	55.56	33.33
	Mar.	0.00	26.67	26.67	46.67

Habitat preferences varied between the two lakes. At Shengjin Lake, cranes preferred mudflats throughout winter (*S* = 0.07 ± 0.06) (Table 2). From November to January, they avoided meadows (*S* = −0.72 ± 0.17) and preferred paddy fields (*S* = 0.32 ± 0.33); however, from February onwards, the avoidance of meadows weakened (*S* = −0.16 ± 0.04) and paddy fields were no longer used. At Caizi Lake, cranes avoided mudflats (*S* = −0.71 ± 0.48) and meadows (*S* = −0.53 ± 0.48). Excluding November, they exhibited a preference for agricultural fields (*S* = 0.69 ± 0.04) and aquaculture ponds (*S* = 0.24 ± 0.33).

**TABLE 2:** Seasonal utilization preferences of foraging habitats for the Hooded Crane at Shengjin and Caizi lakes.

Lakes	Habitats	November	December	January	February	March	Mean	SD
Shengjin Lake	Mudflat	0.22	−0.11	0.03	0.07	0.14	0.07	0.06
	Meadow	−0.75	−0.42	−1.00	−0.12	−0.20	−0.50	0.17
	Paddy	−0.34	0.71	0.59	−1.00	−1.00	−0.21	0.37
Caizi Lake	Mudflat	0.13	−0.86	−0.83	−1.00	−1.00	−0.71	0.21
	Meadow	0.15	−0.23	−1.00	−0.90	−0.65	−0.53	0.22
	Paddy	−1.00	0.64	0.72	0.77	0.61	0.35	0.34
	Aquaculture pond	−1.00	−0.71	0.42	0.48	0.77	−0.01	0.35

The GLMMs results indicated different drivers of foraging population abundance in the two lakes (Table 3). For Shengjin Lake, the best‐fit model (*w*_*i*_ = 0.40) included both biomass and sediment penetrability. Food biomass had a significant positive effect on abundance (*β* = 0.46 ± 0.11, 95% CI: 0.24 to 0.68, *p* < 0.001), while sediment penetrability had a significant negative effect (*β* = −0.64 ± 0.08, 95% CI: −0.80 to −0.48, *p* < 0.001); other factors were not significant (Table 4). For the Caizi Lake, the best‐fit model (*w*_*i*_ = 0.42) retained food biomass, distance to roads, and distance to settlements. Among these, food biomass exerted a highly significant positive effect (*β* = 0.85 ± 0.14, 95% CI: 0.58 to 1.11, *p* < 0.001), while distance to roads had a significant negative effect (*β* = −0.47 ± 0.07, 95% CI: −0.69 to −0.26, *p* < 0.001).

**TABLE 3:** Models of factors influencing foraging hooded crane population abundance.

Lakes	df	LogLik	AICc	ΔAICc	$w_{i}$
Shengjin Lake	6	−499.10	1010.80	0.00	0.40
	7	−498.73	1012.30	1.47	0.19
Caizi Lake	7	−295.00	605.80	0.00	0.42
	8	−294.35	607.00	1.23	0.23
	8	−294.57	607.40	1.68	0.18
	7	−295.92	607.60	1.83	0.17

Abbreviations: ΔAICc, the difference between the AICc value of model *i* and that of the best model; AICc, corrected Akaike Information Criterion; df, degrees of freedom; LogLik, log‐likelihood value; Wi, Akaike weight.

**TABLE 4:** Model‐averaged parameters influencing Hooded Crane habitat use.

Lakes	Influences	Estimate (*β*)	SE	95% CI	RI	VIF	*p*
Shengjin Lake	Food biomass (g/m^2^)	0.46	0.11	0.24, 0.68	1.00	1.53	0.001***
	Food burial depth (cm)	−0.02	0.04	−0.18, 0.07	0.32	1.53	0.695
	Sediment penetrability (N/cm^2^)	−0.64	0.08	−0.80, −0.48	1.00	1.04	0.001***
	Distance to road (m)	0.00	0.03	−0.13, 0.17	0.25	1.98	0.905
	Distance to settlement (m)	0.00	0.03	−0.15, 0.15	0.25	1.99	0.908
Caizi Lake	Food biomass (g/m^2^)	0.85	0.14	0.58, 1.11	1.00	2.47	0.001***
	Food burial depth (cm)	−0.03	0.11	−0.22, 0.11	0.31	1.14	0.68
	Sediment penetrability (N/cm^2^)	0.04	0.08	−0.09, 0.25	0.39	1.23	0.576
	Distance to road (m)	−0.47	0.07	−0.69, −0.26	1.00	2.19	0.001***
	Distance to settlement (m)	0.08	0.06	−0.02, 0.24	0.65	1.28	0.307

*Note:* Estimates indicate the magnitude and direction of the effects of different environmental factors on Hooded Crane, with habitat type and survey session included as random effects. *p* values indicate statistical significance (*** indicates *p* ≤ 0.001).

Abbreviations: 95% CI, 95% confidence interval; RI, relative importance; SE, standard error; *β*, standardized regression coefficient.

## 4 Discussion

Habitat use is influenced by multiple environmental and resource‐related factors (Mott et al. 2023). In wetland ecosystems, water level fluctuations are a primary driver of waterbird habitat use (Wu et al. 2021; Yu et al. 2025), directly controlling the extent of suitable foraging areas and influencing food abundance and accessibility (An et al. 2021; Yu et al. 2025), thereby altering habitat use patterns and population dynamics. Our results demonstrate that water level regulation regimes in gate‐controlled lakes drive distinct habitat use patterns in wintering Hooded Cranes. While cranes at Shengjin Lake predominantly used natural lacustrine habitats (e.g., mudflats), those at Caizi Lake shifted toward artificial habitats (e.g., paddy fields). Food abundance, resource availability, and local environmental conditions were the key drivers of the divergence.

Food abundance and availability are primary drivers of habitat use (Dong et al. 2024; Hou et al. 2025). Our results show that food biomass is likely an important driver of foraging population abundance. At Shengjin Lake, near‐natural water regulation sustained hydrological cycles resembling natural fluctuations. Gradual winter drawdown exposed extensive mudflats and meadows (Palumbo et al. 2019; Zhang et al. 2021), providing cranes with abundant and accessible subterranean tubers that supported the population throughout the wintering period (Zheng et al. 2015). However, the amplitude, frequency, and duration of water level fluctuations significantly modulate the growth rhythms of wetland vegetation (Gao et al. 2020). The distribution and biomass of taxa such as *Carex* are highly sensitive to the rate of water level recession and the timing of exposure (Yuan et al. 2017). Rapid fluctuations or erratic hydrological shifts may have disrupted the normal phenological progression of wetland plants, thereby decoupling the direct linear relationship between water level and meadow area (Huang et al. 2022). In contrast, the artificially high water levels at Caizi Lake likely caused prolonged inundation of plant roots and seeds, suppressed submerged macrophyte growth, and decreased the area of suitable natural habitats (Aharon‐Rotman et al. 2017; Guo et al. 2022; Wei and Zhou 2023). The reduction in food resources showed a positive influence on crane abundance in artificial habitats. Additionally, physical characteristics such as sediment penetrability influence habitat use by mediating food availability (Yu et al. 2025). In natural habitats, high penetration resistance can increase excavation difficulty and limit crane abundance (Wu et al. 2020). However, in artificial habitats, the availability of surface‐scattered rice grains eliminates this constraint. These hydrologically driven differences in food resources and physical conditions underpin the divergent habitat use patterns between the two lakes.

The use of alternative habitats, such as agricultural fields, is a critical strategy for coping with natural habitat loss and degradation (Rajpar et al. 2022). Under intense human disturbance, animals are forced to exploit suboptimal natural habitats or switch to artificial habitats to secure alternative food resources (Godet et al. 2018; Vergin et al. 2025). Agricultural fields have emerged as vital alternative foraging habitats for Hooded Cranes, offering abundant and accessible post‐harvest residues (Zhang et al. 2015; Wan et al. 2016). Beyond paddy fields, aquaculture ponds have also been recognized as significant supplementary foraging habitats (Cheng et al. 2022). The practice of seasonal drawdown for winter harvesting significantly reduces water depth, which not only concentrates prey but also creates extensive artificial mudflats that facilitate high‐density foraging and high intake rates (Fonseca and Navedo 2020; Ji et al. 2024). In Caizi Lake, these managed aquaculture ponds provide essential food resources for Hooded Cranes, offering a critical buffer against the inundation of natural lacustrine habitats. However, these habitats are typically associated with heightened human activity, which can disrupt foraging behavior significantly (Harrison et al. 2018).

In line with the OFT, in animals, there is a trade‐off between energy intake and foraging costs in efforts to maximize net energy gain per unit time (Yang et al. 2015). Our findings from Shengjin Lake support the theory. The reduced use of paddy fields in spring, coinciding with increased anthropogenic disturbance from plowing, reflects a trade‐off between food availability and perceived risk (Lantz et al. 2011). Whereas buried rice grains increase search and handling energy costs, agricultural activities increase disturbance and predation risk. Simultaneously, spring tuber growth in lakeside meadows provides abundant low‐risk food resources (Fox et al. 2011; Fan et al. 2020). Consequently, when natural habitats provide food‐rich and low‐disturbance foraging grounds, cranes exhibit a heightened sensitivity to disturbance in artificial habitats (Palumbo et al. 2019).

At Caizi Lake, the pronounced shift toward artificial habitats under high‐water regulation aligns with the RSFT. The theory predicts that animals with sufficient energy reserves are risk‐averse, favoring constant but lower rewards, whereas those facing energy deficits adopt risk‐prone strategies and seek high‐reward resources despite elevated risks (Caraco et al. 1980; Banerjee and Thaker 2025). Although avoiding human disturbance is generally adaptive, scarcity of natural food at Caizi Lake appears to drive cranes to forage in close proximity to roads despite increased disturbance. The behavioral response parallels that of Black‐necked Cranes ( *Grus nigricollis* ) in the Caohai Wetland, which increasingly tolerate human proximity as food resources diminish (Wu et al. 2020). The habitat shift underscores that in the absence of high‐quality and low‐risk natural habitats, Hooded Cranes will select high‐reward but high‐risk artificial habitats—a critical adaptive trade‐off to meet wintering survival and migratory energy demands amid wetland degradation and resource scarcity (Sung 2015; Wang et al. 2019; Dong et al. 2024).

The effective conservation of wintering waterbirds in gate‐controlled lakes in the middle and lower Yangtze River floodplain is facing unprecedented challenges in the context of non‐natural water level regulation (Liu et al. 2019). To mitigate these impacts, we recommend that hydraulic gate operations be coordinated with the seasonal migratory patterns of waterbirds (Cui et al. 2024). While meeting the lake's water supply demands, management protocols must prioritize the adequate exposure of critical foraging habitats, such as mudflats and meadows (Yu et al. 2025). Furthermore, as natural habitats face prolonged inundation, paddy fields and aquaculture ponds have emerged as vital alternative habitats for Hooded Cranes at Caizi Lake (Cheng et al. 2022). Therefore, fully utilizing and scientifically managing agricultural artificial habitats, particularly the large, contiguous paddy fields surrounding Caizi Lake, can effectively compensate for the loss of food resources caused by natural wetland shrinkage and degradation, thereby sustaining waterbird populations (Rajpar et al. 2022). We propose establishing ecological compensation mechanisms to encourage farmers to retain post‐harvest rice stubble and implementing seasonal closures during the peak wintering period to minimize human activity and traffic interference. These measures will provide a critical buffer for endangered waterbirds, mitigating the loss of natural foraging grounds resulting from altered hydrological regimes.

## 5 Conclusion

Water‐level fluctuations in shallow, gate‐controlled lakes of the middle and lower Yangtze River floodplain are key drivers of foraging habitat‐use patterns in wintering Hooded Cranes. At Shengjin Lake, near‐natural water regulation has enabled sustained use of natural mudflats and meadows. Conversely, at Caizi Lake, artificially high water levels degrade natural foraging habitats, forcing cranes to shift to farmlands and aquaculture ponds as anthropogenic wetlands to secure alternative food resources. Although the shift increased exposure to anthropogenic disturbance, cranes adopted a risk‐prone strategy to balance resource acquisition with survival risk. The adaptive tradeoff highlights how endangered waterbirds cope with wetland degradation under divergent hydrological regimes. Our findings provide a mechanistic basis for the conservation of wintering waterbirds in gate‐regulated lakes across the Yangtze floodplain.

## Author Contributions

**Yong Fang:** data curation (equal), formal analysis (equal), investigation (equal), methodology (equal), software (equal), validation (equal), visualization (equal), writing – original draft (lead). **Yundong Zhong:** data curation (equal), investigation (equal), methodology (equal), software (equal), validation (equal), visualization (equal), writing – review and editing (equal). **Lizhi Zhou:** conceptualization (lead), data curation (equal), funding acquisition (lead), methodology (equal), project administration (lead), resources (lead), supervision (lead), writing – review and editing (equal). **Ming Liang:** formal analysis (equal), methodology (equal), visualization (equal).

## Funding

This work was supported by the National Natural Science Foundation of China (Grant No. 32470553).

## Conflicts of Interest

The authors declare no conflicts of interest.

## Supporting information


**Figure S1:** Comparison of long‐term water level dynamics during the wintering period (December–February) at Shengjin and Caizi lakes from 2014 to 2025.

**Figure S2:** Wintering Hooded Cranes in four typical foraging habitats at the study lakes. (A) Mudflat; (B) Meadow; (C) Paddy field; (D) Aquaculture pond. Photos by the authors, 2024–2025.

## Data Availability

Derived data supporting the findings of this study have been deposited in the Zenodo database (https://zenodo.org/). The data can be accessed at Fang, Yong via the Zenodo Dataset (https://doi.org/10.5281/zenodo.18332640). Additional data related to this study may be requested from the corresponding author upon reasonable request.

## References

[ece373800-bib-0059] Aharon‐Rotman, Y. , J. McEvoy , Z. Zhaoju , et al. 2017. “Water Level Affects Availability of Optimal Feeding Habitats for Threatened Migratory Waterbirds.” Ecology and Evolution 7, no. 23: 10440–10450. 10.1002/ece3.3566. 29238566 PMC5723607

[ece373800-bib-0001] An, L. , K. Liao , L. Zhu , and B. Zhou . 2021. “Influence of River‐Lake Isolation on the Water Level Variations of Caizi Lake, Lower Reach of the Yangtze River.” Journal of Geographical Sciences 31: 551–564. 10.1007/s11442-021-1858-4.

[ece373800-bib-0002] Banerjee, A. , and M. Thaker . 2025. “Risk‐Sensitive Foraging in a Tropical Lizard.” Biology Letters 21, no. 2: 20240628. 10.1098/rsbl.2024.0628. 39965651 PMC11835483

[ece373800-bib-0003] Bi, S. , and L. Zhou . 2025. “The Influence of the Abundance and Availability of Alternative Food on the Foraging Behavior of Wintering Siberian Cranes (*Leucogeranus leucogeranus*).” Avian Research 16: 100254. 10.1016/j.avrs.2025.100254.

[ece373800-bib-0064] Caraco, T. , S. Martindale , and T. S. Whittam . 1980. “An Empirical Demonstration of Risk‐Sensitive Foraging Preferences.” Animal Behaviour 28, no. 3: 820–830. 10.1016/S0003-3472(80)80142-4.

[ece373800-bib-0004] Cheng, B. , X. Zhu , J. M. Alatalo , et al. 2022. “The Impacts of Water Level Fluctuations From Paddy Fields and Aquaculture Ponds on Wetland Habitats for Wintering Waterbirds: Implications for Wetland Management.” Frontiers in Environmental Science 10: 980201. 10.3389/fenvs.2022.980201.

[ece373800-bib-0005] Cui, L. , Z. Wei , L. Zhou , and B. Cheng . 2024. “Effects of Constant High Water Levels in Winter on Waterbird Diversity in Caizi Lakes: A Functional Perspective.” Global Ecology and Conservation 52: e02934. 10.1016/j.gecco.2024.e02934.

[ece373800-bib-0006] Cui, Y. , Y. Tang , S. Yang , et al. 2023. “Changes in Wintering Hooded Cranes and Their Habitats at Chongming Dongtan Over the Past 20 Years.” Avian Research 14: 100083. 10.1016/j.avrs.2023.100083.

[ece373800-bib-0007] Davis, G. H. , M. C. Crofoot , and D. R. Farine . 2022. “Using Optimal Foraging Theory to Infer How Groups Make Collective Decisions.” Trends in Ecology & Evolution 37: 942–952. 10.1016/j.tree.2022.06.010. 35842325

[ece373800-bib-0008] Deng, G. , S. Zhu , H. Jiang , et al. 2023. “Responses of the Siberian Crane Population to Temporal and Spatial Hydrological Variations in Stopover Sites in Northeast China.” Ecological Indicators 154: 110635. 10.1016/j.ecolind.2023.110635.

[ece373800-bib-0009] Dong, H. Y. , H. Wang , Q. M. He , et al. 2024. “The Effects of Temporal and Spatial Variations in Food Resources on the Distribution and Abundance of Black‐Necked Cranes, *Grus nigricollis* .” Ornithology Research 33, no. 11: 5. 10.1007/s43388-024-00214-z.

[ece373800-bib-0010] Elgin, A. S. , R. G. Clark , and C. A. Morrissey . 2020. “Tree Swallow Selection for Wetlands in Agricultural Landscapes Predicted by Central‐Place Foraging Theory.” Condor 122, no. 4. 10.1093/condor/duaa039.

[ece373800-bib-0011] Fan, Y. , L. Zhou , L. Cheng , Y. Song , and W. Xu . 2020. “Foraging Behavior of the Greater White‐Fronted Goose ( *Anser albifrons* ) Wintering at Shengjin Lake: Diet Shifts and Habitat Use.” Avian Research 11, no. 1: 3. 10.1186/s40657-020-0189-y.

[ece373800-bib-0012] Fonseca, J. , and J. G. Navedo . 2020. “Shorebird Predation on Benthic Invertebrates After Shrimp‐Pond Harvesting: Implications for Semi‐Intensive Aquaculture Management.” Journal of Environmental Management 262: 110290. 10.1016/j.jenvman.2020.110290. 32090889

[ece373800-bib-0013] Fox, A. D. , L. Cao , Y. Zhang , Y. Song , and W. Xu . 2011. “Declines in the Tuber‐Feeding Waterbird Guild at Shengjin Lake National Nature Reserve, China—A Barometer of Submerged Macrophyte Collapse.” Aquatic Conservation: Marine and Freshwater Ecosystems 21, no. 1: 82–91. 10.1002/aqc.1154.

[ece373800-bib-0014] Gao, Y. , Y. H. Xie , and D. S. Zou . 2020. “Hydrological Regime Change and Its Ecological Responses in East Dongting Lake, China.” Ecohydrology & Hydrobiology 20, no. 1: 142–150. 10.1016/j.ecohyd.2019.07.003.

[ece373800-bib-0060] Godet, L. , C. Harmange , M. Marquet , E. Joyeux , and J. Fournier . 2018. “Differences in Home‐Range Sizes of a Bird Species in its Original, Refuge and Substitution Habitats: Challenges to Conservation in Anthropogenic Habitats.” Biodiversity and Conservation 27, no. 3: 719–732. 10.1007/s10531-017-1460-3.

[ece373800-bib-0015] Guan, L. , L. Wen , D. Feng , H. Zhang , and G. Lei . 2014. “Delayed Flood Recession in Central Yangtze Floodplains Can Cause Significant Food Shortages for Wintering Geese: Results of Inundation Experiment.” Environmental Management 54, no. 6: 1331–1341. 10.1007/s00267-014-0350-7. 25164981

[ece373800-bib-0016] Guo, W. , Z. Zhou , J. Chen , X. Zheng , and X. Ye . 2022. “Effects of Extreme Flooding on Aquatic Vegetation Cover in Shengjin Lake, China.” Hydrological Processes 36, no. 2: e14459. 10.1002/hyp.14459.

[ece373800-bib-0017] Harrison, A. L. , N. Petkov , D. Mitev , G. Popgeorgiev , B. Gove , and G. M. Hilton . 2018. “Scale‐Dependent Habitat Selection by Wintering Geese: Implications for Landscape Management.” Biodiversity and Conservation 27, no. 1: 167–188. 10.1007/s10531-017-1427-4.

[ece373800-bib-0018] Hou, J. , L. Li , Y. Wang , et al. 2021. “Influences of Submerged Plant Collapse on Diet Composition, Breadth, and Overlap Among Four Crane Species at Poyang Lake, China.” Frontiers in Zoology 18, no. 1: 24. 10.1186/s12983-021-00411-2. 34001190 PMC8130136

[ece373800-bib-0057] Hou, X. , G. Lu , S. Zhang , L. Feng , G. Ren , and H. Wu . 2025. “Terrestrial Nocturnal Roosting Behavior of Black‐necked Cranes (*Grus nigricollis*) on the Yunnan‐Guizhou Plateau: Active Choice or Forced Environmental Adaptation.” Ecology and Evolution 15, no. 6: e71485. 10.1002/ece3.71485. 40519886 PMC12165949

[ece373800-bib-0019] Houston, A. I. , and T. H. Rosenström . 2024. “A Critical Review of Risk—Sensitive Foraging.” Biological Reviews 99, no. 2: 478–495. 10.1111/brv.13031. 37987237

[ece373800-bib-0020] Huang, Y. , X. S. Chen , Y. A. Zou , et al. 2022. “Exploring the Relative Contribution of Flood Regimes and Climatic Factors to *Carex* Phenology in a Yangtze River‐Connected Floodplain Wetland.” Science of the Total Environment 847: 157568. 10.1016/j.scitotenv.2022.157568. 35882330

[ece373800-bib-0021] Huang, Z. , L. Lu , G. Jiao , J. Jiang , and Q. Ye . 2018. “Analysis of the Correlations Between Environmental Factors and Rare Cranes in the Poyang Lake Region of China.” Journal of Great Lakes Research 44, no. 1: 140–148. 10.1016/j.jglr.2017.11.003.

[ece373800-bib-0022] Ji, X. , S. Xia , and L. Zhou . 2024. “Impacts of Reclamation and Aquaculture on the Wintering Waterbird Assemblage at a Floodplain Lakeshore Based on Multidimensional Diversity.” Global Ecology and Conservation 51: e02926. 10.1016/j.gecco.2024.e02926.

[ece373800-bib-0023] Kong, D. , W. Luo , Q. Liu , et al. 2018. “Habitat Use, Preference, and Utilization Distribution of Two Crane Species (*Genus: Grus*) in Huize National Nature Reserve, Yunnan‐Guizhou Plateau, China.” PeerJ 6: e5105. 10.7717/peerj.5105. 30042879 PMC6054782

[ece373800-bib-0063] Lantz, S. M. , D. E. Gawlik , and M. I. Cook . 2011. “The Effects of Water Depth and Emergent Vegetation on Foraging Success and Habitat Selection of Wading Birds in the Everglades.” Waterbirds 34, no. 4: 439–447. 10.2307/41432457.

[ece373800-bib-0024] Lee, O. , G. Gankhuyag , and Y. S. Jo . 2025. “Spatial Ecology of Leopard Cats ( *Prionailurus bengalensis* ) in Korea: Seasonal and Sexual Variation in Home Range and Habitat Preference.” Mammal Research 70: 1–11. 10.1007/s13364-025-00796-z.

[ece373800-bib-0026] Liu, S. Q. , H. W. Tian , S. Ren , et al. 2024. “Social Risk to Infant: The Role of Kin for Maternal Visual Monitoring in Tibetan Macaques.” Ecology and Evolution 14, no. 6: e11626. 10.1002/ece3.11626. 38919651 PMC11196900

[ece373800-bib-0027] Liu, X. W. , H. Q. Li , and Y. Q. Yang . 2019. “Water Level Characteristics of Lake Caizi, Lower Reaches of the Yangtze River During Wintering Period Based on Long‐Term Hydrological Alteration.” [in Chinese.] Journal of Lake Science 31, no. 6: 1662–1669. 10.18307/2019.0606.

[ece373800-bib-0028] Marshall, M. E. , M. L. Morrison , and R. N. Wilkins . 2013. “Tree Species Composition and Food Availability Affect Productivity of an Endangered Species: The Golden‐Cheeked Warbler.” Condor 115, no. 4: 882–892. 10.1525/cond.2013.130013.

[ece373800-bib-0056] Mott, R. , T. A. Prowse , M. V. Jackson , et al. 2023. “Measuring Habitat Quality for Waterbirds: A Review.” Ecology and Evolution 13, no. 4: e9905. 10.1002/ece3.9905. 37038530 PMC10082184

[ece373800-bib-0058] Palumbo, M. D. , S. A. Petrie , M. Schummer , B. D. Rubin , and S. Bonner . 2019. “Mallard Resource Selection Trade‐Offs in a Heterogeneous Environment During Autumn and Winter.” Ecology and Evolution 9, no. 4: 1798–1808. 10.1002/ece3.4864. 30847073 PMC6392399

[ece373800-bib-0029] Rajpar, M. N. , S. Ahmad , M. Zakaria , et al. 2022. “Artificial Wetlands as Alternative Habitat for a Wide Range of Waterbird Species.” Ecological Indicators 138: 108855. 10.1016/j.ecolind.2022.108855.

[ece373800-bib-0030] Shen, D. , F. Qian , S. Xia , et al. 2025. “Impacts of Prolonged Dry Season and Artificial Food Supply on the Wintering Spatial Distribution of Siberian Cranes: Implications for Conservation.” Avian Research 16: 100308. 10.1016/j.avrs.2025.100308.

[ece373800-bib-0065] Sung, C. Y. 2015. “Simulation of Crane Habitat Fragmentation in the North and South Korean Border Region After Korean Reunification.” Landscape and Urban Planning 134: 10–18. 10.1016/j.landurbplan.2014.10.008.

[ece373800-bib-0031] Ucero, A. , I. Abril‐Colón , C. Palacín , J. M. Álvarez‐Martínez , and J. C. Alonso . 2025. “Nest‐Site and Brood‐Rearing Habitat Selection in Canarian Houbara Bustards: The Importance of Concealment and Food Availability.” Journal of Ornithology 166, no. 2: 399–413. 10.1007/s10336-024-02224-6.

[ece373800-bib-0032] Vergin, L. , K. K. Clausen , and J. Madsen . 2025. “The Role of Winter Cereals as Cold‐Weather Refuge for Taiga Bean Geese *Anser fabalis fabalis* Wintering in Denmark.” Journal of Ornithology 166: 803–814. 10.1007/s10336-025-02262-8.

[ece373800-bib-0033] Wachu, C. M. , B. M. Mwangi , J. Jumbe , W. Wamiti , and J. Mwangi . 2025. “Seasonal Habitat Shifts and Wetland Dependence of Grey Crowned Crane in Urbanising Kenya.” African Journal of Ecology 63, no. 6: e70084. 10.1111/aje.12738.

[ece373800-bib-0062] Wan, W. , L. Zhou , and Y. Song . 2016. “Shifts in Foraging Behavior of Wintering Hooded Cranes (*Grus monacha*) in Three Different Habitats at Shengjin Lake, China.” Avian Research 7, no. 1: 13. 10.1186/s40657-016-0047-0.

[ece373800-bib-0066] Wang, C. , B. Dong , M. Zhu , et al. 2019. “Habitat Selection of Wintering Cranes (Gruidae) in Typical Lake Wetland in the Lower Reaches of the Yangtze River, China.” Environmental Science and Pollution Research 26, no. 8: 8266–8279. 10.1007/s11356-019-04306-. 30706266

[ece373800-bib-0034] Wang, C. , S. Xia , X. Yu , L. Wen , and C. Y. Choi . 2026. “Artificial Wetlands as Drought Refugia for Wintering Geese: Adaptive Use or Ecological Mismatch?” Biological Conservation 313: 111545. 10.1016/j.biocon.2025.111545.

[ece373800-bib-0035] Wang, W. , J. D. Fraser , and J. Chen . 2017. “Wintering Waterbirds in the Middle and Lower Yangtze River Floodplain: Changes in Abundance and Distribution.” Bird Conservation International 27, no. 2: 167–186. 10.1017/s0959270915000398.

[ece373800-bib-0036] Wang, X. Y. , B. Jiang , Z. F. Tian , J. Z. Cai , and G. J. Lin . 2018. “Impact of Water Level Changes in Lake Caizi (Anhui Province) on Main Wetland Types and Wintering Bird Habitat During Wintering Period.” [in Chinese.] Journal of Lake Science 30, no. 6: 1636–1645. 10.18307/2018.0615.

[ece373800-bib-0037] Wang, Y. , X. Ji , and L. Zhou . 2024. “Functional Diversity Dynamics of Waterbird Communities Driven by Water Levels at Shengjin Lake, a Small River‐Connected Shallow Lake in the Middle and Lower Yangtze River Floodplain.” Ecology and Evolution 14, no. 10: e70222. 10.1002/ece3.70222. 39376473 PMC11456756

[ece373800-bib-0038] Wei, Z. , and L. Zhou . 2023. “The Impact of Earlier Flood Recession on Metacommunity Diversity of Wintering Waterbirds at Shallow Lakes in the Middle and Lower Yangtze River Floodplain.” Avian Research 14: 100102. 10.1016/j.avrs.2023.100102.

[ece373800-bib-0039] Wu, D. , C. Hu , M. Zhang , Z. Li , and H. Su . 2020. “Foraging Habitat Selection of Overwintering Black‐Necked Cranes in the Farming Area Surrounding the Caohai Wetland, Guizhou Province, China.” Avian Research 11, no. 1: 5. 10.1186/s40657-020-00192-y.

[ece373800-bib-0040] Wu, H. , J. Dai , S. Sun , et al. 2021. “Responses of Habitat Suitability for Migratory Birds to Increased Water Level During Middle of Dry Season in the Two Largest Freshwater Lake Wetlands of China.” Ecological Indicators 121: 107065. 10.1016/j.ecolind.2020.107065.

[ece373800-bib-0055] Xu, P. , S. Mao , S. Zhang , G. Bempah , and Y. Zhao . 2024. “Habitat Utilization of the Eurasian Spoonbill (*Platalea leucorodia*) Wintering in the Yancheng National Nature Reserve: Relative Importance of Artificial Habitats.” Frontiers in Ecology and Evolution 12: 1357765. 10.3389/fevo.2024.1357765.

[ece373800-bib-0041] Xu, P. , X. Zhang , F. Zhang , et al. 2020. “Use of Aquaculture Ponds by Globally Endangered Red‐Crowned Crane ( *Grus japonensis* ) During the Wintering Period in the Yancheng National Nature Reserve, a Ramsar Wetland.” Global Ecology and Conservation 23: e01123. 10.1016/j.gecco.2020.e01123.

[ece373800-bib-0042] Xu, Z. , B. Dong , X. Gao , et al. 2022. “Research on Ecological Security of Shengjin Lake Wetland (Anhui Province of China) Based on TM Images.” Journal of the Indian Society of Remote Sensing 50, no. 6: 1087–1099. 10.1007/s12524-022-01510-1.

[ece373800-bib-0043] Yang, L. , L. Zhou , and Y. Song . 2015. “The Effects of Food Abundance and Disturbance on Foraging Flock Patterns of the Wintering Hooded Crane ( *Grus monacha* ).” Avian Research 6, no. 1: 15. 10.1186/s40657-015-0024-z.

[ece373800-bib-0044] Yao, J. , J. Gao , X. Yu , and Q. Zhang . 2022. “Impacts of a Proposed Water Control Project on the Inundation Regime in China's Largest Freshwater Lake (Poyang Lake): Quantification and Ecological Implications.” Journal of Hydrology: Regional Studies 40: 101024. 10.1016/j.ejrh.2022.101024.

[ece373800-bib-0045] Yu, F. , J. Zhai , Z. Huang , J. Chen , F. Han , and L. Wang . 2025. “The Impact of Poyang Lake Water Level Changes on the Landscape Pattern of Wintering Wading Bird Habitats.” Global Ecology 58: e03453. 10.1016/j.gecco.2025.e03453.

[ece373800-bib-0046] Yuan, S. , Z. Yang , X. Liu , and H. Wang . 2017. “Key Parameters of Water Level Fluctuations Determining the Distribution of *Carex* in Shallow Lakes.” Wetlands 37, no. 6: 1005–1014. 10.1007/s13157-017-0934-0.

[ece373800-bib-0061] Zhang, D. , L. Zhou , and Y. Song . 2015. “Effect of Water Level Fluctuations on Temporal‐Spatial Patterns of Foraging Activities by the Wintering Hooded Crane (*Grus monacha*).” Avian Research 6, no. 1: 16. 10.1186/s40657-015-0026-x.

[ece373800-bib-0047] Zhang, Y. , L. Zhou , L. Cheng , and Y. Song . 2021. “Water Level Management Plan Based on the Ecological Demands of Wintering Waterbirds at Shengjin Lake.” Global Ecology and Conservation 27: e01567. 10.1016/j.gecco.2021.e01567.

[ece373800-bib-0048] Zhao, F. , L. Zhou , and W. Xu . 2013. “Habitat Utilization and Resource Partitioning of Wintering Hooded Cranes and Three Goose Species at Shengjin Lake.” Chinese Birds 4, no. 4: 281–290. 10.5122/cbirds.2013.0032.

[ece373800-bib-0049] Zheng, M. , L. Zhou , N. Zhao , and W. Xu . 2015. “Effects of Variation in Food Resources on Foraging Habitat Use by Wintering Hooded Cranes ( *Grus monacha* ).” Avian Research 6: 11. 10.1186/s40657-015-0020-3.

[ece373800-bib-0050] Zheng, X. , J. Chen , W. Guo , Z. Zhou , and X. Ye . 2022. “Response of Phytoplankton Community Structure to Vegetation Restoration After Removal of Purse Seine in Shengjin Lake.” Diversity 14, no. 3: 178. 10.3390/d14030178.

[ece373800-bib-0054] Zhi, Y. , Y. Cao , J. Sun , W. Li , and E. Jeppesen . 2018. “Indirect Effects of Extreme Precipitation on the Growth of *Vallisneria denseserrulata* Makino.” Environmental and Experimental Botany 153: 229–235. 10.1016/j.envexpbot.2018.06.003.

[ece373800-bib-0051] Zhong, Y. , L. Cheng , Y. Fan , L. Zhou , and Y. Song . 2022. “The Foraging Window for Greater White‐Fronted Goose ( *Anser albifrons* ) is Consistent With the Growth Stage of *Carex* .” Diversity 14, no. 11: 943. 10.3390/d14110943.

[ece373800-bib-0052] Zhou, S. , A. Krzton , S. Gao , C. Guo , and Z. Xiang . 2021. “Effects of Human Activity on the Habitat Utilization of Himalayan Marmot ( *Marmota himalayana* ) in Zoige Wetland.” Ecology and Evolution 11, no. 13: 8957–8968. 10.1002/ece3.7733. 34257938 PMC8258216

[ece373800-bib-0053] Zhu, Y. , H. Wang , and W. Guo . 2021. “The Impacts of Water Level Fluctuations of East Dongting Lake on Habitat Suitability of Migratory Birds.” Ecological Indicators 132: 108277. 10.1016/j.ecolind.2021.108277.

