# Supplementary material for: Water Level Regulation Regime Shifts Drive Divergent Foraging Habitat Use by Wintering Hooded Cranes (Grus monacha) in Shallow Gate‐controlled Lakes of the Yangtze Floodplain
Source: Ecol Evol. 2026 Jun 9;16(6):e73800. doi: 10.1002/ece3.73800 (PMC13249527; doi:10.1002/ece3.73800)
Supplement: Supplementary file 1 — Figure S1: Comparison of long‐term water level dynamics during the wintering period (December–February) at Shengjin and Caizi lakes from 2014 to 2025. [file ECE3-16-e73800-s001.docx]

**Supporting Information：**





**Fig. S1.** Comparison of long-term water level dynamics during the wintering period (December–February) at Shengjin and Caizi lakes from 2014 to 2025.
