# Supplementary material for: Water Level Regulation Regime Shifts Drive Divergent Foraging Habitat Use by Wintering Hooded Cranes (Grus monacha) in Shallow Gate‐controlled Lakes of the Yangtze Floodplain
Source: Ecol Evol. 2026 Jun 9;16(6):e73800. doi: 10.1002/ece3.73800 (PMC13249527; doi:10.1002/ece3.73800)
Supplement: Supplementary file 2 — Figure S2: Wintering Hooded Cranes in four typical foraging habitats at the study lakes. (A) Mudflat; (B) Meadow; (C) Paddy field; (D) Aquaculture pond. Photos by the authors, 2024–2025. [file ECE3-16-e73800-s002.docx]

**Supporting Information：**


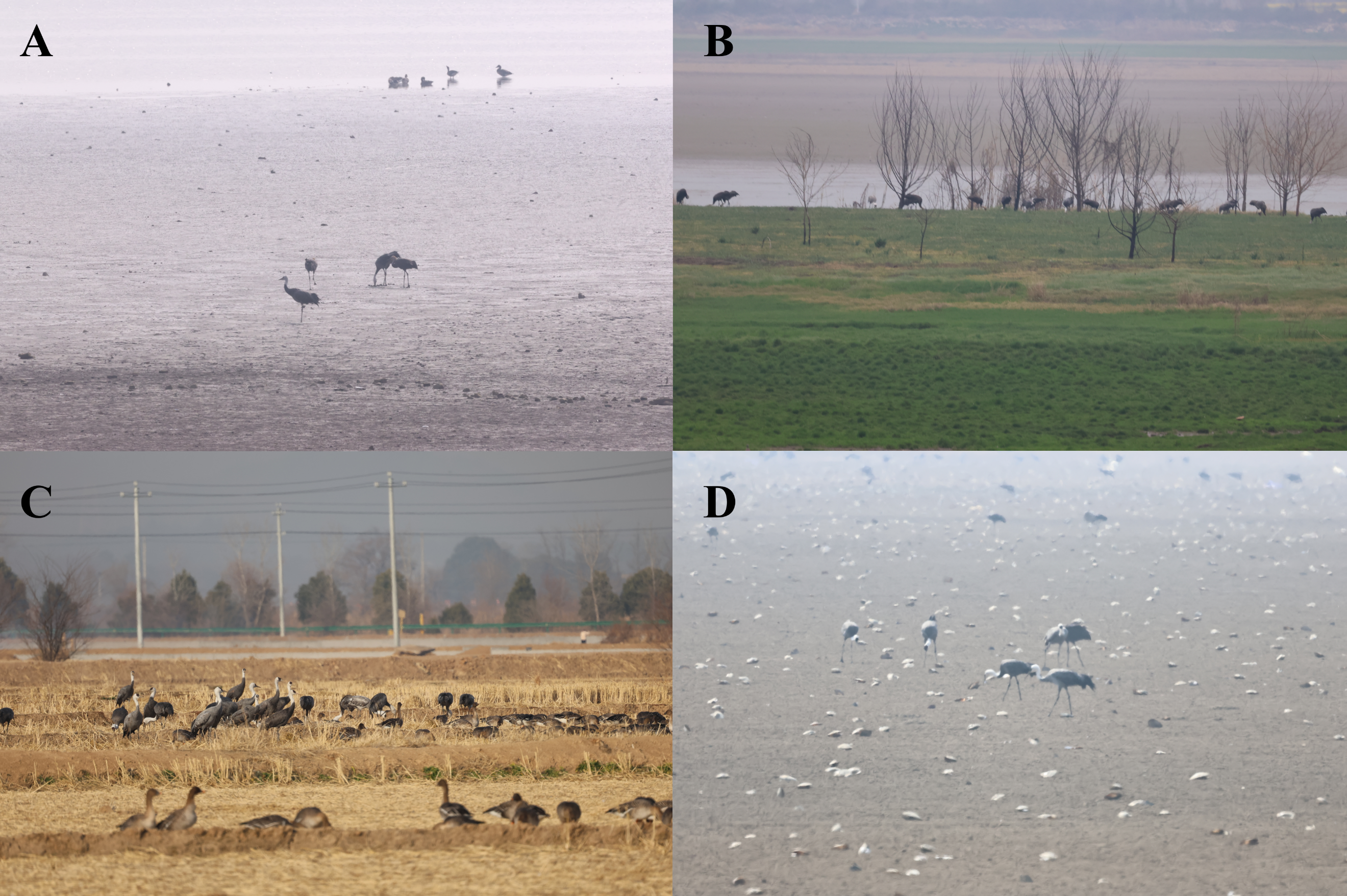


**Fig. S2.** Wintering Hooded Cranes in four typical foraging habitats at the study lakes. (A) Mudflat; (B) Meadow; (C) Paddy field; (D) Aquaculture pond. Photos by the authors, 2024–2025.
